# *Sparganothis sulfureana* (Lepidoptera: Tortricidae) egg surface characteristics stimulate parasitism by *Ascogaster mimetica* (Hymenoptera: Braconidae)

**DOI:** 10.1093/jisesa/ieae092

**Published:** 2024-09-07

**Authors:** Yahel Ben-Zvi, Cesar Rodriguez-Saona

**Affiliations:** Department of Entomology, Rutgers University, New Brunswick, NJ, USA; Department of Entomology, Rutgers University, New Brunswick, NJ, USA

**Keywords:** *Vaccinium macrocarpon*, Sparganothis fruitworm, *Choristoneura parallela*, scales, host recognition

## Abstract

*Ascogaster mimetica* Viereck is an egg-larval parasitoid that targets *Sparganothis sulfureana* Clemens, a major cranberry pest in North America. While previous studies have shown that other *Ascogaster* species respond to cues from their hosts’ eggs, it remains unknown whether *A. mimetica* utilizes these cues to recognize *S. sulfureana*. We hypothesized that female *A. mimetica* recognizes *S. sulfureana* from another cranberry pest, *Choristoneura parallela* Robinson, based on the presence of surface cues on eggs. To test this, we observed female *A. mimetica* behavior when exposed to eggs from its host, *S. sulfureana*; a nonhost, *C. parallela*; and eggs of *S. sulfureana* that were washed clean with hexane. Additionally, we tracked parasitism rates in each group. Our results revealed that *A. mimetica* spent 9.5 times longer walking when exposed to *C. parallela* eggs and 6 times longer when exposed to hexane-washed *S. sulfureana* eggs compared to unwashed *S. sulfureana* eggs. Also, *A. mimetica* spent 3 times longer grooming when exposed to hexane-washed than unwashed *S. sulfureana* eggs. In contrast, females spent 6 and 18 times longer drumming and probing/ovipositing on unwashed *S. sulfureana* eggs than on *C. parallela* eggs and 5 times longer probing/ovipositing on unwashed *S. sulfureana* eggs than on hexane-washed *S. sulfureana* eggs. Higher parasitism rates were observed from unwashed *S. sulfureana* eggs compared to those from *C. parallela* eggs and hexane-washed *S. sulfureana* eggs. Our findings suggest that the presence of egg surface cues, like scales, of *S. sulfureana* likely plays a crucial role in host acceptance and parasitism success for *A. mimeti*ca.

Parasitoids often rely on various cues to locate and accept host species, thereby facilitating successful parasitism of suitable hosts (Vinson 1976, Turlings et al. 1990, Vet and Dicke 1992). Many of these cues, known as kairomones, are associated with the host (Vet and Dicke 1992, Seino and Kainoh 2008), including scales shed from the bodies of lepidopterous hosts onto eggs during oviposition (DeLury et al. 1999, Vargas et al. 2020). For instance, female *Ascogaster reticulata* Watanabe (Hymenoptera: Braconidae), which parasitizes the smaller tea tortrix *Adoxophyes* sp. (Lepidoptera: Tortricidae), uses scales from its host moth to locate host eggs (Kainoh et al. 1990). Similarly, *Ascogaster quadridentata* Wesmael females are attracted to kairomones released from scales deposited on eggs by its host, the codling moth *Cydia pomonella* L. (Lepidoptera: Tortricidae) (DeLury et al. 1999).

In cranberries (*Vaccinium macrocarpon* Aiton), Sparganothis fruitworm (*Sparganothis sulfureana* Clemens) (Lepidoptera: Tortricidae) and spotted fireworm (*Choristoneura parallela* Robinson) (Lepidoptera: Tortricidae) rank among the top insect pests in North America (Ben-Zvi and Rodriguez-Saona 2023). In New Jersey (USA), these tortricids share similar life cycles, completing 2 generations per year and overwintering as early instar larvae (de Lange and Rodriguez-Saona 2015a, 2015b). Adults become active during bloom, mate soon after, and then lay eggs around fruit set (de Lange and Rodriguez-Saona 2015a, 2015b). The larvae from the second generation are the most damaging since they feed on fruit, causing yield losses. The most common parasitoid of *S. sulfureana* in New Jersey is the egg-larval parasitoid *Ascogaster mimetica* Viereck (C.R-S. unpublished data). As an egg-larval parasitoid, *A. mimetica* oviposits into host eggs, and the developed parasitoids emerge from late-instar host larvae (Kainoh and Tamaki 1982). Besides *S. sulfureana*, *A. mimetica* is only known to attack the strawberry leafroller, *Ancylis comptana* Frölich (Lepidoptera: Tortricidae) (Shaw 1983). The mechanism by which *A. mimetica* accepts its host *S. sulfureana* and avoids the nonhost *C. parallela* remains unknown.

This study aimed to investigate the influence of tortricid species (host versus nonhost) and the presence of cues on the host eggs on the behaviors and parasitism rates of *A. mimeti*ca. Our hypothesis was that female *A. mimetica* recognizes its host eggs, *S. sulfureana*, from the nonhost, *C. parallela*, based on egg surface cues, like scales. To test this, we conducted observations of female *A. mimetica* behaviors when presented with 3 types of eggs: those of *S. sulfureana*, *C. parallela*, or *S. sulfureana* eggs washed clean with hexane. In addition to behavioral observations, we tracked the eggs from these 3 groups to assess parasitism rates.

## Materials and Methods

### Insect Rearing

Egg masses of *S. sulfureana* and *C. parallela* used in behavioral and parasitism studies were obtained from laboratory-grown colonies that originated from larvae collected in commercial cranberry bogs in Chatsworth, NJ (USA). These colonies were maintained for several generations with new field-collected material added yearly, and the larvae were reared on Stonefly Heliothis Diet (Ward’s Scientific, Rochester, NY, USA). To collect eggs for rearing and experiments, 5-8 males and females were placed inside 20.5 cm × 46 cm plastic bags, and the eggs laid on the bags were collected. The colony of *A. mimetica* was established from parasitized *S. sulfureana* larvae also collected from commercial cranberry bogs in Chatsworth, NJ, and maintained for fewer than 5 generations on *S. sulfureana*. Adult *A. mimetica* were reared in a BugDorm Insect Rearing cage (30 cm × 30 cm × 30 cm) (BioQuip Inc., Rancho Dominguez, CA, USA) and provided with 10% honey-water. All colonies were maintained in separate incubators set at 25 ± 1 °C, with a relative humidity of 65%, and a 14:10 L:D photoperiod at the Rutgers Marucci Center (Chatsworth, NJ, USA).

### Behavioral Observations

A gravid (2–3 days old) *A. mimetica* female, which had not been exposed to eggs since emerging, was placed in a polystyrene Petri dish (10 cm diameter, 1.5 cm height) (Sigma-Aldrich, St. Louis, MO, USA) along with one (<48 h old) egg mass of *S. sulfureana* (*N* = 15), *C. parallela* (*N* = 14), or *S. sulfureana* rinsed in 1 ml of 99.9% hexane (Fisher Scientific, Hampton, NH, USA) for 10 s to remove surface cues such as scales (*N* = 14). Successful removal of scales was confirmed under a stereomicroscope (Nikon SMZ-U, Tokyo, Japan). Each egg mass contained ~60 eggs and was affixed to the bottom of the Petri dish using tape.

The behaviors of *A. mimetica* both on and off the eggs were recorded for 60 min using The Observer software (ver. 5.0; Noldus Information Technology, Wageningen, Netherlands). This observation period aligns with previous oviposition behavior studies conducted on *A. reticulata* (Kainoh et al. 1990). Behaviors recorded while off the eggs included walking (time spent moving around the Petri dish), tapping (touching the surface of the Petri dish with antennae), antennating (waving antennae in an elevated position), standing still (time spent resting without contacting the egg mass), and grooming (cleaning antennae). Behaviors recorded while on the eggs included drumming (touching the egg mass with antennae), standing still (time spent resting in contact with the egg mass), and probing/ovipositing (touching an egg with the ovipositor and potentially ovipositing).

### Parasitism Rates

In a separate study with different individuals, egg masses of *S. sulfureana* (*N* = 10), *C. parallela* (*N* = 6), and hexane-washed *S. sulfureana* (*N* = 10) were exposed to a naïve, gravid (2–3 days old) female *A. mimetica* for 60 min in a Petri dish as described above. After exposure to the parasitoid, the eggs were incubated at 25 ± 1 °C, 65% relative humidity, and a 14:10 L:D photoperiod. We recorded the total egg count per mass, emerged larvae, and emerged parasitoid adults. Parasitism rates were calculated by dividing the total number of emerged parasitoids by the total egg count.

### Statistical Analyses

For the behavioral study, we calculated the percentage of time that female *A. mimetica* spent performing each behavior. Because the response variable was the proportion of time spent on each behavior, we arcsine square-root transformed the data prior to analysis. Our explanatory variable was the 3 egg types: *S. sulfureana*, *C. parallela*, and *S. sulfureana* washed with hexane. Since the behaviors are not independent, we first conducted a Permutational Multiple Analysis of Variance (PERMANOVA) using RStudio (ver. 4.2.1; R Core Team 2022) with the “vegan” package and “adonis2” function (Oksanen et al. 2024). Following, to identify specific behaviors influenced by the type of eggs, we performed Kruskal–Wallis nonparametric tests on each individual behavior. If significant, we used Holm’s adjusted P-value with pairwise Wilcoxon’s post hoc tests.

Based on the preliminary test where females of *A. mimetica* were exposed to either 20 µl of hexane (*N* = 3) or a blank control (*N* = 2), the behaviors recorded did not show significant effects of hexane on *A. mimetica* behavior (*F* = 1.862; *df* = 1,4; *P* = 0.4), as assessed by PERMANOVA. Moreover, hexane is a solvent commonly used in studies investigating parasitoid behavior (e.g., Tognon et al. 2016).

We similarly arcsin square-root transformed the parasitism rates and analyzed the data, along with the egg count, emerged larvae, and emerged parasitoids, using Kruskal–Wallis nonparametric tests followed by Holm’s adjusted *P*-value with pairwise Wilcoxon’s post hoc tests. Replicates without emerged larvae were removed from the analysis because for an egg-larval parasitoid, an unemerged larva would mean unsuccessful parasitism.

## Results

### Behavioral Observations

**Fig. 1.**
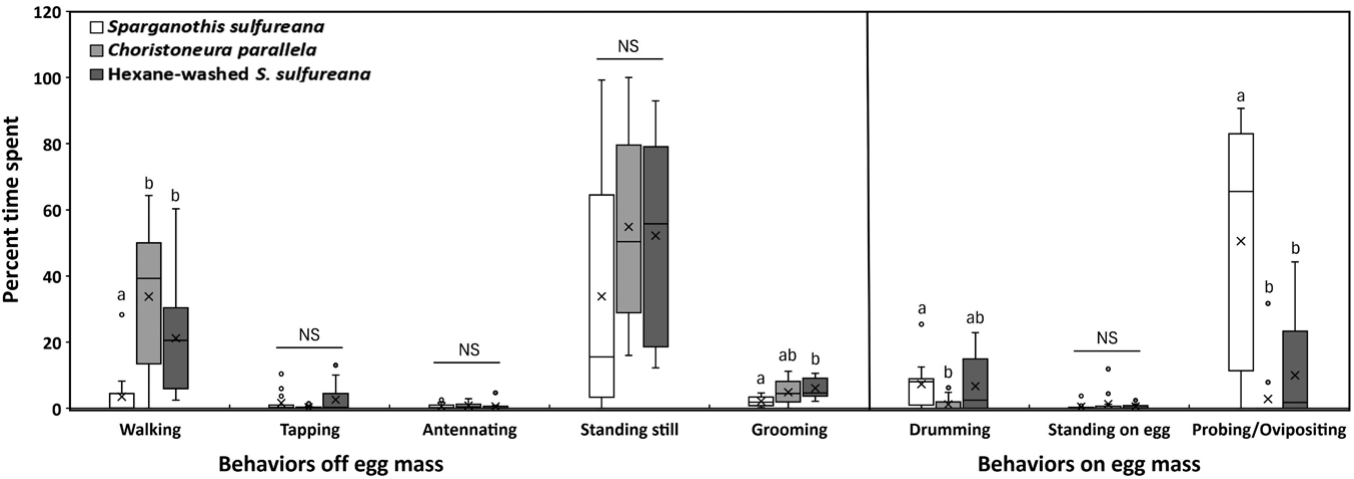
Box plots of percent of time that *Ascogaster mimetica* spent performing behaviors off and on the different egg mass types: *Sparganothis sulfureana*, *Choristoneura parallela*, and hexane-washed *S. sulfureana*. For each box plot, the X mark represents the mean, the line is the median, box borders are the interquartile range (IQR) (25th–75th percentiles), and the whiskers represent minimal and maximal values in the range of 1.5 IQR; dots represent outliers beyond the 1.5 IQR. Different letters represent significant differences among egg mass types within each behavior (pairwise Wilcoxon’s test, *P* < 0.05); NS = nonsignificant (pairwise Wilcoxon’s test, *P* > 0.05).

There were significant differences among the behaviors of female *A. mimetica* when exposed to *S. sulfureana*, *C. parallela*, and hexane-washed *S. sulfureana* egg masses (*F* = 8.535; *df* = 2,40; *P* = 0.001). Among the behaviors off the egg masses, *A. mimetica* spent 9.5 and 6 times longer walking when exposed to *C. parallela* and hexane-washed *S. sulfureana* egg masses, respectively, than when exposed to *S. sulfureana* egg masses (*χ*^2^ = 16.787; *df* = 2; *P* < 0.001; Fig. 1). Also *A. mimetica* spent 3 times longer grooming when exposed to hexane-washed *S. sulfureana* egg masses than to unwashed *S. sulfureana* egg masses (*χ*^2^ = 13.323; *df* = 2; *P* = 0.001; Fig. 1). Among the behaviors on the egg masses, *A. mimetica* spent 6 times longer drumming on *S. sulfureana* egg masses than on *C. parallela* egg masses (*χ*^2^ = 8.905; *df* = 2; *P* = 0.012), and 18 and 5 times longer probing/ovipositing on *S. sulfureana* egg masses than on *C. parallela* and hexane-washed *S. sulfureana* egg masses, respectively (*χ*^2^ = 17.215; *df* = 2; *P* < 0.001; Fig. 1). All other behaviors were nonsignificant (*P* > 0.05; Fig. 1).

### Parasitism Rates

Although the number of eggs per mass did not differ among egg types, the eclosion rate of *S. sulfureana* eggs was 4.5 times higher than that of *C. parallela* eggs. The parasitism rates of *A. mimetica* were significantly higher on unwashed egg masses of *S. sulfureana* compared to those washed with hexane. None of the *C. parallela* eggs were parasitized by *A. mimetica* (Table 1).

**Table 1.** Mean number (± SE) of eggs per mass, emerged larvae, and emerged *Ascogaster mimetica* parasitoids, as well as percent parasitism from *Sparganothis sulfureana* egg masses, *Choristoneura parallela* egg masses, and *S. sulfureana* egg masses washed with hexane

Type of egg mass	No. eggs per mass	No. emerged larvae	No. emerged parasitoids	% Parasitism
*Sparganothis sulfureana*	64.9 ± 9.4^a^	56.8 ± 10.8^a^	17.2 ± 4.2^a^	26.7 ± 5.3^a^
*Choristoneura parallela*	60.3 ± 9.8^a^	11.2 ± 7.5^b^	0.0 ± 0.0^b^	0.0 ± 0.0^b^
Hexane-washed *S. sulfureana*	58.3 ± 4.3^a^	45.8 ± 5.4^a^	2.2 ± 1.0^b^	3.6 ± 1.5^b^
*χ* ^2^	0.196	9.483	14.264	14.412
*df*	2	2	2	2
*P*	0.907	0.009	<0.001	<0.001

Different letters within columns represent significant differences among egg mass types (pairwise Wilcoxon’s test, *P* < 0.05).

## Discussion

The oviposition behavior and parasitism rates of *A. mimetica* were compared between egg masses of its host with scales attached versus eggs rinsed with hexane to remove the scales. The no-choice tests also included eggs of the nonhost *C. parallela*. The *A. mimetica* females showed greater oviposition activity and achieved higher parasitism on untreated *S. sulfureana* eggs. Interestingly, *A. mimetica* exhibited similar behaviors on *C. parallela* and the treated *S. sulfureana* eggs, suggesting that the parasitoid ceased to recognize host eggs after a hexane wash, perceiving them as nonhosts. Compared to the intact and washed *S. sulfureana* eggs, *C. parallela* had significantly lower larval emergence and produced no parasitoids, indicating that oviposition by the parasitoid may have killed both or that no oviposition occurred.

**Fig. 2.**
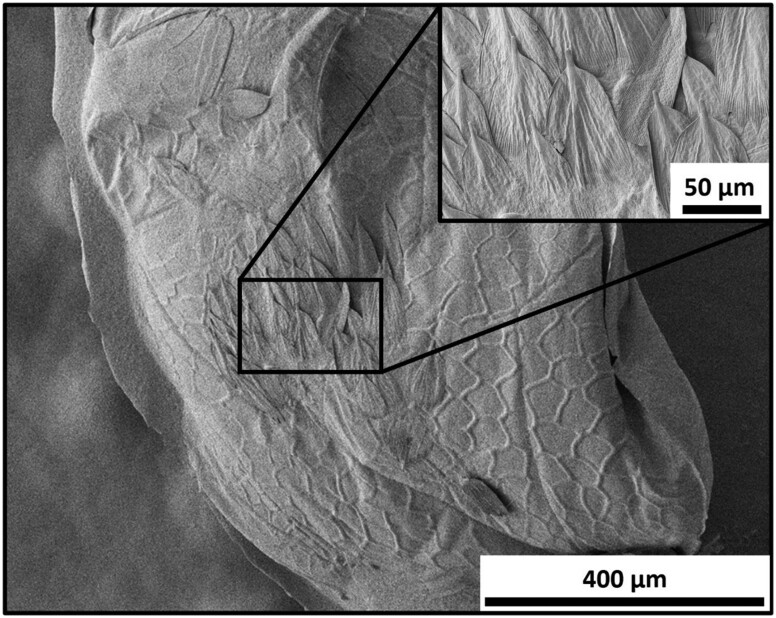
Scanning electron microscope image of a *Sparganothis sulfureana* egg mass with scales. The egg mass was dry mounted, and the images were taken at 3.1 pA, 1 kV, and between 2.99 E-5 and 3.16 E-5 Pa.

This study indicated that behavioral cues on the egg surface, including scales from ovipositing *S. sulfureana* females (Fig. 2), are required for host acceptance and parasitism by *A. mimeti*ca. The hexane wash removed the scales and possibly other cues, such as kairomones and surface texture, which facilitate host acceptance by the parasitoid (Kainoh and Tamaki 1982, Kainoh et al. 1990, DeLury et al. 1999). Elimination of these cues resulted in a parasitism rate of *A. mimetica* similar to the nonhost *C. parallela*. Thus, short-range tactile and possibly chemical cues enable *A. mimetica* to distinguish between eggs of *S. sulfureana* and *C. parallela* in the same environment.

Our studies were conducted using lab-reared colonies in laboratory conditions; therefore, field confirmation is essential as *A. mimetica* may rely on additional cues in their natural environment. In natural settings, *A. mimetica* can encounter both *S. sulfureana* and *C. parallela*, and likely utilizes volatiles from plants as long-range cues. For example, while both *S. sulfureana* and *C. parallela* are important cranberry pests, *C. parallela* also feeds and oviposits on weeds like leatherleaf (Stuart and Polavarapu 1998), which might be avoided by *A. mimeti*ca. Understanding the cues that parasitoids respond to and whether they can be conditioned to these cues is crucial for effective biological control. For instance, *A. reticulata* can distinguish between different plants consumed by its host *Adoxophyes honmai* Yasuda and can associate certain plants with the presence of the moth if conditioned (Kawakami and Kainoh 1986, Seino and Kainoh 2008). Although the *A. mimetica* colony used in our experiments was reared on *S. sulfureana*, we used unconditioned individuals. Therefore, follow-up studies should determine if conditioning *A. mimetica* to egg kairomones improves their efficacy as biocontrol agents.

In conclusion, *A. mimetica* utilizes egg surface cues, such as scales, of *S. sulfureana* to recognize them as suitable hosts. When *S. sulfureana* eggs are hexane-washed, *A. mimetica* treats them similarly to a nonhost, *C. parallela*, resulting in reduced parasitism rates. Given that both *S. sulfureana* and *C. parallela* are active in the same environment simultaneously, egg surface cues like scales likely provide crucial short-range chemical and tactile information to the wasp during host selection. Future research should focus on identifying volatiles emitted by the scales of *S. sulfureana* that attract *A. mimetica*, as well as further exploring the correlation between *A. mimetica* oviposition behavior and parasitism rates.
